# Self-reported attitudes, skills and use of evidence-based practice among Swiss chiropractors: a national survey

**DOI:** 10.1186/s12998-022-00462-0

**Published:** 2022-12-20

**Authors:** Arlette Albisser, Petra Schweinhardt, André Bussières, Mirjam Baechler

**Affiliations:** 1 Faculty of Medicine, University of Zurich, Zurich, Switzerland; 2 Department of Chiropractic Medicine, Balgrist University Hospital, University of Zurich, Zürich, Switzerland; 3 Département Chiropratique, Université du Québec à, Trois-Rivières, Canada; 4 School of Physical and Occupational Therapy, Faculty of Medicine and Health Sciences, McGill University, Québec, Canada

**Keywords:** Evidence-based practice, Chiropractic, Survey, Knowledge translation, Dissemination and implementation

## Abstract

**Study Objectives:**

The high burden of disease associated with musculoskeletal disorders severely impacts patients’ well-being. As primary care providers, Swiss chiropractors ought to contribute towards identifying and using effective treatment strategies. An established approach is the full integration of evidence-based practice (EBP). This study aimed to investigate the attitudes, skills and use of EBP among Swiss chiropractors, as well as investigating potential facilitators and barriers for its adoption.

**Methods and material:**

All 329 members of the Swiss Association of Chiropractic (ChiroSuisse) were invited in March 2021 to participate in this cross-sectional survey. Data were acquired anonymously online, using the Evidence-Based practice Attitude and utilization SurvEy (EBASE). The survey encompassed 55 questions measuring attitudes (n = 8, response range 1–5; total score range of 8–40), skills (n = 13, response range 1–5; total score range of range of 13–65) and use of EBP (n = 6, response range 0–4; total score range of 0–24).

**Results:**

228 (69.3%) chiropractors returned complete EBASE questionnaires. This sample was representative of all ChiroSuisse members with respect to gender, age groups and proportion of chiropractic residents. Respondents generally held positive attitudes towards EBP, as indicated by the high mean (31.2) and median (31) attitude sub-score (range 11–40). Self-reported skills had a mean sub-score of 40.2 and median of 40 (range 13–65). Knowledge about EBP-based clinical practice had been primarily obtained in chiropractic under- or postgraduate education (33.8% and 26.3%, respectively). Use of EBP achieved a lower sub-score, with mean and median values of 7.4 and 6, respectively (range 0–24). The most commonly identified barriers preventing EBP uptake were lack of time (67.9%) and lack of clinical evidence in chiropractic/manual therapy-related health fields (45.1%).

**Conclusion:**

Swiss chiropractors held favourable attitudes and reported moderate to moderate-high skill levels in EBP. Nevertheless, similar to chiropractors in other countries, the self-reported use of EBP was relatively low, with lack of time and lack of clinical evidence being the most named barriers.

## Introduction

The high burden of disease associated with musculoskeletal disorders (MSD) strains health care systems worldwide [1–3]. In fact, MSD ranked top twenty (place 19) regarding disability-adjusted life-years (DALYs) in 2019, when compared to 329 other diseases and injuries [4]. Low back pain (LBP) and neck pain (NP) in particular are among the top ten global leading causes of disability contributing to the need for rehabilitation [3, 5, 6].

MSD, including LBP and NP, are the main focus of chiropractors as primary care providers. Being one of the five government-recognized medical professions in Switzerland, chiropractic carries a lot of responsibility to use effective treatment strategies [7–10].

An established approach in the medical community to address the increasing burden of MSD is improved patient treatment through the full integration of evidence-based practice (EBP). The key ideas of using “current best evidence from clinical research in the management of individual patients” dates back as far as the renaissance [11–19]. Since then, the potential of EBP to effectively manage health disorders including LBP and NP has increased tremendously. The three pillars of EBP are best available research evidence, clinical expertise and patients’ values and preferences [11–13, 20–24]. Modern technologies provide virtually unlimited access to scientific research from all around the world [18]. Despite these advances, the clinical application of research results and corresponding guidelines remains challenging [18, 22, 25–27]. Significant determinants for the uptake of research results are perceptions, attitudes and beliefs regarding EBP [28]. For example, it was found that limited uptake of EBP is often caused by a misunderstanding of the concept [28]. Thus, further investigation is required to not only determine the current attitudes, skills and use of EBP among chiropractors, but also to identify potential barriers and facilitators for its full integration into everyday clinical practice.

The World Federation of Chiropractic, the European Council on Chiropractic Education (ECCE) and the Swiss Association of Chiropractic (ChiroSuisse) have all defined the delivery of evidence-based care as one of the main goals in their strategy or as an educational outcome [29–31]. Studies conducted in Sweden, Canada and the U.S. [7, 9, 32–40] have investigated how chiropractors include research evidence in their clinical practice. In general, study participants showed favourable attitudes towards EBP and believed that it supports their decision-making [9, 32–36, 40]. However, these investigations also identified insufficient knowledge transfer into practice [9, 32–35].

In Switzerland, studies investigating the attitudes towards EBP and recording its level of implementation have been conducted among nurses and allied health care providers [41–48]. The results are comparable to the studies cited above, with generally positive attitudes towards EBP but unsatisfactory implementation into clinical practice [43, 46]. Analyses focusing on Swiss chiropractors are currently missing.

Therefore, the objectives of this study were to investigate the attitudes, skills and use of EBP among Swiss chiropractors, and to identify potential facilitators and barriers toward EBP adoption in clinical practice.

## Methods

### General

This national online survey was conducted between March 30, 2021 and July 11, 2021. The survey was administered anonymously online through REDCap, a secure web-based application [49].

This study was exempted from ethics review by the ethic commission of Kanton Zürich, as it did not fall within the scope of Swiss Federal Human Research Act (BASEC-Nr. Req-2021-00,173).

### Participants and recruitment

In March 2021, all members of the professional association of chiropractors in Switzerland, ChiroSuisse, i.e. 296 fully qualified Swiss chiropractors and 33 chiropractic residents (postgraduates undergoing mandatory training, similar to pursuing a medical specialty, before being eligible for licensing), were sent an individualized link to the survey in REDCap (56). To ensure blinding, all communication with the study participants was performed by an independent member of the research team at the Department of Chiropractic Medicine at Balgrist University Hospital.

To achieve a high response rate, two advance notices were provided before launching the survey at the end of March 2021. Following the Dillman survey method [50, 51], non-respondents were reminded to participate in predefined intervals. In the present study, a total of six reminder emails were sent, one every other week. Furthermore, an appeal to join the study was made by the authors and through email by the president of ChiroSuisse. As an incentive to participate, all respondents were awarded three continuing education points upon completion of the survey.

### Material

To achieve the study aim, a national online survey based on the Evidence-Based practice Attitude and utilization SurvEy (EBASE) questionnaire was conducted among Swiss chiropractors in 2021. According to methods described by Polit and Hungler, this questionnaire is a self-administered tool with acceptable content and convergent validity, and good test–retest reliability (Cronbach’s alpha ≥ 0.84) [52–54]. EBASE has already been used in similar studies among chiropractors worldwide [9, 32–35]. Permission to use the tool was obtained from one of the developers in November 2020 through personal communication.

The original EBASE questionnaire consists of six parts (Part A–Part F) with a total of 64 questions, as well as a demographic section. Parts A, B and D focus on the attitudes, skills and use respectively, each generating a sub-score proportional to how strong the answers are in favour of EBP. To allow international comparison, the content and scoring rubric of these three sections was not modified and the original survey language (English) was kept. The questions in Part C aim to measure the level of EBP-related training and education. The original questions required adaptation to the Swiss under- and postgraduate chiropractic education program [8, 55]. Finally, common barriers and facilitators are investigated in Parts E and F, respectively. Part E was extended with a question to select the top three barriers from a given list and some statements in Part F were combined to improve readability. In addition, minor modifications in the wording based on Schneider and colleagues [32] and Bussières and colleagues [32] were adopted to make the survey more suitable for chiropractors.

In view of future studies, two more sections (Part G and H) were included. Part G asked about the x-ray behaviour and general awareness of guidelines, which is an important measure for use of EBP in daily practice not covered by the three EBASE sub-scores. A non-existing (i.e. thoracic) guideline was added to identify potential social-desirability bias. Part H focussed on the chiropractor’s role and identity [7, 56, 57]. The other results of Parts G and H, are outside the scope of this paper and will be published separately. The survey encompassed a total of 81 questions and was pilot tested before deployment, in order to rule out misunderstandings and verify the time needed for completion of approximately 20 min.

### Data analysis

Survey data were analysed using IBM® SPSS® Statistics for Windows, Version 27 (Armonk, New York, IBM Corp.). Representativeness of the sample with respect to the ChiroSuisse member base was verified in terms of gender, age groups and proportion of chiropractic residents using the one sample chi-square test. Descriptive and inferential statistics were calculated for each item in Parts A, B, D, E and F, including response frequencies and mean for normally distributed data, as well as both mean and median for non-normally distributed values. Sub-scores of attitudes (Part A), skills (Part B) and use (Part D) were computed by summing the first eight items of Part A (response range 1–5, score range 8–40), all 13 items of Part B (response range 1–5, score range 13–65) and the first six items of Part D (response range 0–4, score range 0–24) [52]. Higher sub-scores indicate higher self-reported levels of attitudes, skills and use of EBP. Possible associations between demographic variables, the three sub-scores and recognition of the three EBP pillars were explored using Kendall’s Tau correlation coefficient. The coefficients were interpreted as follows: weak correlation (0.10–0.29), moderate correlation (0.30–0.49) and strong correlation (0.50–1.00) [58–60].

## Results

### Demographics

In total, 228 out of the 329 invited chiropractors and chiropractic residents completed the entire survey (response rate of 69.3%). The gender, age group distribution as well as the proportion of chiropractic residents did not significantly differ from the data provided by ChiroSuisse (p’s > 0.13). Apart from chiropractic, 43.9% of the participants obtained an additional higher education or even a postgraduate degree (6.1%) or PhD (0.9%). Nearly two thirds (65.4%) had been in practice for over 16 years, and 44.3% shared a clinic with other chiropractors.

The detailed results for the demographic section are provided in Table 1.

**Table 1:** Baseline demographics of 228 Swiss chiropractors who completed the whole online survey

Variable	Characteristics	N	(%)
Gender	Male	137	(60.1)
	Female	91	(39.9)
Age	Mean (Range)	48.6	(26–80)
	20–29 years	15	(6.6)
	30–39 years	45	(19.7)
	40–49 years	66	(28.9)
	50–59 years	57	(25.0)
	60–65 years	27	(11.8)
	> 65 years	18	(7.9)
Years in practice since fully-licensed	Less than 2 years	11	(5.3)
	2–4 years	23	(11.1)
	5–15 years	38	(18.3)
	16–25 years	70	(33.7)
	More than 25 years	66	(31.7)
Fully-licensed chiropractor		186	(89.0)
Chiropractic residents		19	(8.3)
Highest education level other than chiropractic	Bachelor or equivalent	35	(14.6)
	Master or equivalent	45	(19.7)
	PhD	2	(0.9)
	Postgraduate degree	14	(6.1)
	None	128	(56.1)
	Other	15	(6.6)
Role in clinic	Individual practitioner	78	(34.2)
	One of two or more chiropractors	101	(44.3)
	Practitioner in multi-disciplinary setting	45	(19.7)
	Other	4	(1.8)
Patients seen per week	Less than 50	19	(8.3)
	50–99	86	(37.7)
	100–149	77	(33.8)
	150–199	19	(8.3)
	200–249	17	(7.5)
	250–300	6	(2.6)
	More than 300	4	(1.8)
Onsite X-ray machine (in their clinic)		146	(64.0)

### Attitudes toward EBP

In general, the participants showed positive attitudes toward EBP and agreed or strongly agreed (> 70%) with seven of the ten attitude statements of EBP. Also, nearly all subjects (93.9%) did not feel that the adoption of EBP places an unreasonable demand on their practice or were at least neutral with respect to the statement. The majority of respondents (80.3%) were interested to learn more about EBP. Accordingly, the mean (31.18) and median (31) of the attitudes sub-score were high (range 11–40). These values are consistent with EBASE-based studies amongst chiropractors in Canada, the U.S. and Sweden (Table 10 in the appendix).

While many chiropractors (75.4%) agreed or strongly agreed that clinical experience is part of the decision-making process in EBP, just about half (48.2%) answered that a patient’s preference also has to be taken into account for EBP. Respondents who disagreed with either statement showed a weak negative correlation with respect to both skills (τ = − 0.239, p = 0.001) and use (τ = − 0.162, p = 0.004) sub-scores.

A weak negative correlation between the attitudes sub-score and age (τ = − 0.117, p = 0.005), as well as years in practice since fully-licensed (τ = − 0.128, p = 0.009) was found across the entire sample. Additionally, a weak positive correlation was observed between the attitudes sub-score and the highest degree apart from chiropractic (τ = 0.158, p = 0.002).

A quantitative overview of the responses in the attitude part is shown in Table 2.

**Table 2:** Part A - Self-reported attitudes toward the listed statements

	Strongly Disagree (%)	Disagree (%)	Neutral (%)	Agree (%)	Strongly agree (%)
*1. Evidence-based practice is necessary in the practice of chiropractic	1.3	1.3	7.9	53.1	36.4
*2. Professional literature (e.g. articles, journals & textbooks) and research findings are useful in my day-to-day practice	0.4	2.2	15.8	58.8	22.8
*3. I am interested in learning or improving the skills necessary to incorporate evidence-based practice into my practice	0.4	3.5	15.8	54.4	25.9
*4. Evidence-based practice improves the quality of my patient’s care	1.3	4.4	16.7	54.4	23.2
*5. Evidence-based practice assists me in making decisions about patient care	1.3	1.8	13.2	57.5	26.3
*6. Evidence-based practice takes into account my clinical experience when making clinical decisions	0.9	6.1	17.5	48.2	27.2
*7. Evidence-based practice takes into account a patient’s preference for treatment	3.1	17.1	31.6	30.7	17.5
*8. The adoption of evidence-based practice places an unreasonable demand on my practice	11.4	41.2	41.2	4.8	1.3
9. There is a lack of evidence from clinical trials to support most of the treatments I use in my practice	5.3	44.7	29.8	18.4	1.8
10. Prioritizing evidence-based practice within chiropractic practice is fundamental to the advancement of the profession	0.4	8.8	20.2	48.2	22.4

*belongs to the attitudes sub-score

These are responses to the question “On a scale ranging from “strongly disagree” to “strongly agree”, how would you rate your opinion on the following statements? (please select the best answer for each category)”

### Skills in EBP

Participants were confident in their ability to identify knowledge gaps and answerable clinical questions, reporting skill levels of 4 and 5 in 61% or 65.7% of the cases. That being said, lack of expertise in conducting clinical research and systematic reviews became apparent. Specifically, a poor rating (1 or 2) was selected by 75.5% of the subjects in the former statement and by 68.8% in the latter. Table 3 displays the complete list of results. Evaluating the self-reported skills resulted in a mean sub-score of 40.2 with median at 40 (range 13–65). As shown in Table 10, these values are slightly lower compared to international scores.

**Table 3:** Part B - Self-reported skills level in the listed areas

	1 poor (%)	2 (%)	3 (%)	4 (%)	5 advanced (%)
*11. Identifying knowledge gaps in practice	0.4	5.3	33.3	53.5	7.5
*12. Identifying answerable clinical questions	1.8	5.3	27.2	53.9	11.8
*13. Locating professional literature (e.g. journal articles)	4.4	14.0	32.0	37.3	12.3
*14. Online database searching (e.g. PubMed/Medline)	7.5	16.7	31.1	31.1	13.6
*15. Retrieving evidence	5.7	14.9	42.1	30.7	6.6
*16. Critical appraisal of evidence	3.1	17.1	42.1	32.9	4.8
*17. Synthesis of research evidence	4.8	20.2	41.2	30.7	3.1
*18. Applying research evidence to patient cases	2.6	14.5	32.0	44.7	6.1
*19. Sharing evidence with colleagues	3.9	23.2	32.0	32.5	8.3
*20. Conducting clinical research (e.g. clinical trials)	39.5	36.0	16.7	7.0	0.9
*21. Using findings from clinical research	4.8	11.4	44.7	35.1	3.9
*22. Conducting systematic reviews	34.2	34.6	25.0	5.3	0.9
*23. Using findings from systematic reviews	8.3	19.7	38.6	28.5	4.8

*belongs to the skills sub-score

These are responses to the question “On a scale from 1 to 5, with 1 being poor and 5 being advanced, how would you rate your skills in the following areas? (please select one per skill area)”

Similar to the attitudes sub-score, the skills sub-score was weakly negatively correlated with age (τ = − 0.119, p = 0.004)) and years in practice since fully-licensed (τ = − 0.101, p = 0.029). A weak positive correlation was found between the skills sub-score and the highest degree (τ = 0.197, p = 0.0001). No statistically significant associations were found with respect to other demographic factors.

### Training in EBP

For most participants, knowledge about EBP-based clinical practice had been primarily obtained in chiropractic under- or postgraduate education, with 33.8% and 26.3% respectively. Informal personal study was reported as the main source by 13.6%. Responses to all predefined statements are shown in Table 4. Additional statements in “Other” included: yearlong practice, student examination, discussion and exchange with other practitioners and common sense. Only 2.2% of the participants had not received any training. This group exhibited a weak negative correlation with both the attitudes sub-score (τ = − 0.153, p = 0.006) and skills sub-score (τ = − 0.266, p = 0.001).

**Table 4:** Part C - Self-reported setting in which the most in-depth training in the listed topic was received

	None (%)	Informal personal study (%)	Seminar (< 1 day) (%)	Short course (< 1 week) (%)	Specific course (> 1 week) (%)	Chiropractic (undergraduate) education (%)	Postgraduate education (Institute/Academy) (%)	Postgraduate degree (%)	Other (please specify) (%)
24. Evidence-based clinical practice/evidence-based chiropractic	2.2	13.6	8.8	5.7	2.2	33.8	26.3	4.8	2.6
25. Applying research evidence to clinical practice	4.8	21.9	5.3	9.6	1.8	23.2	24.6	5.7	3.1
26. Critical thinking/critical analysis	3.9	21.5	6.6	4.8	2.2	28.1	21.5	6.1	5.3

These are responses to the question “Please indicate in what setting you have received the most in-depth training in the following areas (please select the best answer for each category)”

### Use of EBP

Relatively conservative mean and median sub-score values of 7.4 and 6 (range 0–24) were achieved for the use of EBP (Table 10 in the appendix). Still, 56.1% of the participants stated that at least half of their practice is based on clinical research evidence. Over 93% reported to use professional literature related to the practice at least once a month, even though 36.8% also stated that it did not change their clinical practice at all. Layperson books and websites of non-government institutions, as well as online databases were not used in the last month by 56.6% and 41.2% of the respondents, respectively. A complete overview of all responses is provided in Table 5.

**Table 5:** Part D - Self-reported use frequency of the listed activities over the last month

	Never	1–5 times	6–10 times	11–15 times	16 + times	
*27. I have read/reviewed professional literature (e.g. professional journals & textbooks) related to my practice	6.6%	56.1%	22.8%	5.3%	9.2%	
*28. I have read/reviewed clinical research findings related to my practice	17.5%	61.0%	8.8%	6.6%	6.1%	
*29. I have used professional literature or research findings to assist my clinical decision-making	12.7%	61.8%	13.6%	7.5%	4.4%	
*30. I have used professional literature or research findings to change my clinical practice	36.8%	51.8%	7.5%	1.8%	2.2%	
*31. I have used an online database (e.g. PubMed/Medline, Cochrane, CINAHL) to search for practice-related literature or research	41.2%	38.6%	9.6%	6.1%	4.4%	
*32. I have used an online search engine (e.g. Google) to search for practice-related literature or research	11.0%	47.8%	25.4%	7.5%	8.3%	
33. I have consulted a chiropractic colleague to assist my clinical decision-making	27.6%	46.1%	16.7%	5.7%	3.9%	
34. I have consulted a colleague from another healthcare profession to assist my clinical decision-making	16.7%	64.9%	10.5%	4.8%	3.1%	
35. I have referred to magazines, layperson/self-help books, or non-government/non-education institution websites to assist my clinical decision-making	56.6%	34.6%	4.4%	2.2%	2.2%	
Proportion	None (0%)	Very small (1–25%)	Small (26–50%)	Moderate (51–75%)	Large (76–99%)	All (100%)
36. What percentage of your practice do you estimate is based on clinical research evidence (i.e. evidence from clinical trials)?	0.9%	17.1%	25.9%	37.7%	18.4%	0.0%

* belongs to the use sub-score

These are responses to the question “Please indicate how often you have performed the following activities over the last month (please select the best answer for each category)”

A weak positive correlation was found between the use sub-score and the highest degree obtained (τ = 0.117, p = 0.017). Statistically significant associations with other demographic factors were not observed.

### Barriers and facilitators to EBP uptake

Lack of time (67.9%) and lack of clinical evidence in chiropractic/manual therapy-related health fields (45.1%) were often judged as being moderate or major barriers preventing EBP uptake by the participants (Table 6). These results are also reflected in the answer to the additional question (“please select up to three top barriers from the provided list that prevent you most from participating evidence-based practice"), where lack of time (65.4%), lack of clinical evidence in chiropractic/manual therapy-related health fields (42.1%) and lack of relevance to chiropractic practice (25.4%) were commonly rated among the top three most restricting barriers. Chiropractors selecting lack of time as the most important barrier are more likely to apply at least one of the guidelines presented in Part G in daily practice, when compared to respondents stating another barrier (τ = 0.134, p = 0.043).

**Table 6:** Part E - Self-reported barriers preventing from participation in EBP

	Not a barrier (%)	A minor barrier (%)	A moderate barrier (%)	A major barrier (%)	Selected among top three barriers* (%)
37. Lack of time	10.5	21.5	44.7	23.2	65.4
38. Lack of resources (i.e. access to a computer, the internet or online databases)	61.8	23.2	13.2	1.8	8.8
39. Lack of clinical evidence in chiropractic/manual therapy-related health fields	17.5	37.3	34.6	10.5	42.1
40. Insufficient skills for locating research	36.4	39.9	18.9	4.8	18.9
41. Insufficient skills for interpreting research	36.4	36.8	21.5	5.3	15.8
42. Insufficient skills to critically appraise/evaluate the literature	36.0	36.0	22.8	5.3	20.2
43. Insufficient skills to apply research findings to clinical practice	37.7	41.2	18.0	3.1	11.4
44. Lack of incentive to participate in evidence-based practice	32.0	36.8	22.8	8.3	17.1
45. Lack of interest in evidence-based practice	53.1	25.0	14.9	7.0	12.7
46. Lack of relevance to chiropractic practice	44.7	29.4	20.2	5.7	25.4
47. Lack of peer support for evidence-based practice	39.0	41.7	16.7	2.6	10.1
48. Patient preference for treatment	41.2	37.7	17.5	3.5	19.3

These are responses to the question “On a scale ranging from "not a barrier" to “major barrier", to what extent do the following factors prevent you from participating in evidence-based practice?”

*This column displays how often the respective barrier was selected to be among the top three barriers preventing the participation in EBP

Regarding potential facilitators (Table 7), all but one of the listed enablers were rated as moderately or very useful by over 80% of the subjects. The most reported resources were free access to online databases (85.1%), as well as access to download full-text journal articles (82.5%) and access to the internet at the workplace in general (79.9%). The only statement that was rated low was “access to tools used to assist the critical appraisal”, which 28.1% thought was only slightly or not useful at all.

**Table 7:** Part F - Self-reported facilitators assisting in participation in EBP

	Not useful	Slightly useful	Moderately useful	Very useful
50. Access to the internet in your workplace	15.4%	4.8%	16.7%	63.2%
51. Free access to online databases that usually require license fees (e.g. DynaMed, CINAHL, Amboss, Orthobullets, Surf, UpToDate)	4.8%	10.1%	17.1%	68.0%
52. Access to download full-text/full-length journal articles	3.9%	13.6%	19.3%	63.2%
53. Access to online education materials related to evidence-based practice	2.6%	11.0%	29.8%	56.6%
54. Access to tools used to assist the critical appraisal/evaluation of research evidence	5.3%	22.8%	36.0%	36.0%
55. Access to critically appraised research papers/topics relevant to your field (e.g. RRS-Education = Research-Review-Education)	3.9%	14.5%	28.5%	53.1%

These are responses to the question “On a scale ranging from "not useful" to "very useful", to what extent would the following strategies assist you in participating in evidence-based practice?”

### Awareness and application of evidence-based guidelines

When asked to choose from a given list, most participants were aware of low back pain (90.4%), neck pain (78.1%) and x-ray guidelines (73.2%). Only 6% of the participants did not recognise any of the provided options, as listed in Table 8. These distributions were similar when looking at the actual application of the guidelines (Table 8). Low back pain, neck pain and x-ray guidelines were used the most in the last month, namely by 69.3%, 59.6% and 48.2% of the chiropractors. In general, 60% of the participants stated that their overall patient treatment had been influenced by a guideline, mostly regarding the details of the treatment (e.g. duration, frequency) or use of x-ray. Participants who were aware of the guideline tend to be aware of more guidelines overall (τ = 0.621, p = 0.000). Similarly, application of the thoracic guideline is moderately positively correlated (τ = 0.490, p = 0.000) to the total number of applied guidelines.

**Table 8:** Part G - Self-reported awareness and application of listed evidence-based guidelines

Guidelines	N		N	
	Aware		Applied	
Low back pain	206 (%)	(90.4) (%)	158 (%)	(69.3) (%)
Thoracic pain	58 (%)	(25.4) (%)	30 (%)	(13.2) (%)
Neck pain	178 (%)	(78.1) (%)	136 (%)	(59.6) (%)
Extremities	46 (%)	(20.2) (%)	26 (%)	(11.4) (%)
X-Ray	167 (%)	(73.2) (%)	110 (%)	(48.2) (%)
None	13 (%)	(5.7) (%)	44 (%)	(19.3) (%)
Other (please specify)	11 (%)	(4.8) (%)	4 (%)	(1.8) (%)

These are responses to the questions “Are you aware of any existing evidence-based guidelines for the following (please select all that apply)” and “In the last month, did you apply any evidence-based guidelines for the following (please select all that apply)”

### Role and identity

Inspired by the work of McGregor and colleagues and Gislason and colleagues [7, 39], the first four statements (Table 9) correspond to an orthodox view, while the fifth choice represents an unorthodox perspective to evidence-based care and guidelines. Although the latter was selected rarely (2.6%, i.e. 6 chiropractors), a statistically significant weak negative correlation was found between an unorthodox view and the skills sub-score (τ = − 0.135, p = 0.007). No other statistically significant connections were observed.

**Table 9:** Part H - Self-reported view of most predominant treated conditions

	N	(%)
I treat musculoskeletal and neuromusculoskeletal problems and include specific disorders such as but not limited to low back and neck related pain	160	(70.2)
I treat the broadest spectrum of health concerns and may include lifestyle and wellness issues	31	(13.6)
I treat vertebral subluxation as a somatic joint dysfunction and/or related to functional or musculoskeletal problems	17	(7.5)
I treat a combination of biomechanical and organic/visceral complaints	14	(6.1)
I treat vertebral subluxation as an encumbrance to the expression of health—vertebral subluxation is seen as an entity	6	(2.6)

These are responses to the question “Which of the following statements best describes the predominant view you have of the conditions you treat?”

## Discussion

This is the first national survey investigating the self-reported attitudes, skills and use of EBP among Swiss chiropractors. A response rate of 69.3% was achieved and the participants were representative of the members of ChiroSuisse. Generally, Swiss chiropractors showed favorable attitudes towards EBP and reported moderate to moderate-high skill levels in EBP. Nevertheless, the provided answers suggest only a low to moderate use of EBP, with lack of time being stated as the most prominent barrier. Overall, these results are in line with other EBP studies among chiropractors [9, 32, 33], as discussed in detail in the following sections.

### Attitudes towards EBP

The attitudes sub-score was high and also consistent with studies conducted in Canada, the U.S. and Sweden [9, 32, 33]. However, a significant number of the Swiss respondents did not recognise two of the three pillars of EBP, namely taking patient’s preference into account (50%) and considering clinical experience (25%). Interestingly, this same group showed a weak negative correlation with respect to both skills sub-scores and use sub-scores. This means that one can apply EBP despite not fully understanding the meaning of EBP.

Another noteworthy relationship was the weak negative correlation between the attitudes sub-score and age and years in practice across all participants. The demographical information indicated that over 30% of the respondents have been working for more than 25 years, meaning they completed their degree in the 1990’s the latest. Although the concept of EBP was already developed at the time, its uptake accelerated in the past 20 years, supported by new technologies, increased research effort and focused education [18, 61–64]. This seems to be reflected in the answers of younger chiropractors with fewer years in practice providing higher attitudes sub-scores.

### Skills in EBP

The self-reported EBP skills resulted in mean and median sub-score values of 40 out of 65, corresponding to moderate to moderate-high skill levels. Compared to international scores, the Swiss results are slightly lower [9, 32, 33]. Multiple factors might be responsible for this difference. First, the lower response rate in the other studies (4.4–33% compared to 69.3% in the present study) increases the likelihood of a sampling bias. Although our sample was representative of the profession with respect to age groups, gender and proportion of chiropractic residents, a participation bias favouring EBP cannot be ruled out [65, 66]. In other words, chiropractors with less EBP skills and usage may have returned incomplete surveys or decided not to participate in the first place. Another reason for the lower score of Swiss chiropractors could be the so-called imposter phenomenon, resulting in lower self-assessments compared to the true skill levels [67]. However, future research is needed to confirm whether such a tendency for underestimation is more prevalent among the Swiss respondents compared to survey participants in other countries.

Despite the difference in sub-score, the Swiss and other international studies identified the lack of expertise in conducting clinical research or systematic reviews. Given that most chiropractors are primarily practitioners and not researchers, this is not surprising and indicates that skills-related questions need to be chosen carefully in future studies, depending on whether the focus lies on EBP in research or in clinical practice.

### Training in EBP

As expected, chiropractors without any training in EBP showed a weak negative correlation to both the attitudes sub-score and skills sub-score. However, no correlation to the use sub-score was observed, which is somewhat counterintuitive. It appears that one either does not require training to use EBP or, perhaps more likely, the use sub-score might not be valid. This point is discussed in more detail in the next section.

### Use, barriers and facilitators of EBP

A low to moderate EBP use sub-score was observed among Swiss chiropractors similar to studies from Canada, the U.S. and Sweden [9, 32, 33]. These results are consistent with other health care professions in Switzerland (studies not based on EBASE), also concluding a positive attitude but poor implementation and use of EBP [41, 43, 46]. Similar factors as for the skills sub-score, i.e. non-response bias in the other studies and underestimation of one’s skills in Switzerland, may explain the lower average scores observed in the present study.

Nonetheless, more than half of the Swiss chiropractors stated that at least half of their practice is based on clinical research. This is partly supported by the results in part G, where two-thirds of the participants indicated being aware of multiple different guidelines, with nearly 50% reporting having applied the low back pain, neck pain or x-ray guidelines in the last month. Over half of the respondents (60%) reported their practice behaviour being influenced by guidelines. With this contradiction, the question arises whether the use sub-score accurately captures the integration level of EBP in everyday clinical practice. For example, although the EBASE questionnaire inquires about searching, reading and discussing professional literature, there is no guarantee that the knowledge gained is applied when treating patients. In other words, the application of guidelines is not captured in the use sub-score, even though it might be a relevant measure for daily use of EBP. Indeed, it appears as if being more involved in research or receiving more research training is prioritised in the current use sub-score formulation, as suggested by the weak positive correlation between the score and the highest additional degree besides chiropractic. In summary, future studies should include questions more relevant for implementing EBP in clinical practice to help determine the need to revise the use sub-score.

Although the informative value of the use sub-score might be limited, conclusions can still be drawn with respect to common barriers and facilitators. Lack of time was the most frequently reported barrier. However, the effect on the implementation of clinical research in daily practice might not be particularly high, as indicted by a weak positive correlation between selecting this particular barrier compared to another one and the application of guidelines.

### Role and identity

Studies by McGregor and colleagues and Gislason and colleagues [7, 39] concluded that unorthodox views are associated with opposition to or even contravention of EBP and less frequent application of guidelines. In the present study, only 6 out of the 228 participants held an unorthodox view according to the definition suggested by Gislason and colleagues [7]. Thus, the weak negative correlation to the skills sub-score has to be treated with caution.

### Study strengths and limitations

A high response rate of 69.3% was achieved and the respondents were representative of all ChiroSuisse members in terms of gender, age groups and proportion of chiropractic residents. Although the EBASE questionnaire was extended and adapted for this survey, the sub-scores computation remained unchanged, allowing comparison with other studies using the same survey.

Two major limitations can be identified, namely survey fatigue and social-desirability bias.

An indication for survey fatigue is given by the fact that a total of 17 people dropped-out before completing the survey. Different efforts were made to reduce this effect, including: (1) administering a single survey instead of multiple shorter ones, (2) measuring and communicating the time required to complete the survey beforehand, allowing participants to schedule a time slot specifically for answering the questions and (3) providing incentive (continuing education points) for completion of the survey [68–70].

Finally, a potential social-desirability bias could be identified in part G. When asked to select known guidelines, 25% of the participants stated that they are aware of a thoracic guideline and 13% claimed to be applying it in practice. However, this response choice was purposely added to challenge the participants, as no such guideline exists to the best of the authors’ knowledge. Thus, this answer might have been selected because some respondents wanted to display the “desirable” behaviour of knowing and applying as many guidelines as possible. This argument is supported by the strong/moderate positive correlation between being aware/applying thoracic guideline and being aware/applying more guidelines overall. Self-reported surveys are prone to social-desirability bias and this assessment provides some information about its magnitude in the present study [71].

## Conclusion

This is the first study about EBP attitudes, skills and use among Swiss chiropractors. Swiss chiropractors held favourable attitudes and reported moderate to moderate-high skill levels in EBP. Nevertheless, similar to chiropractors in other countries, the self-reported use of EBP was relatively low, with lack of time and lack of clinical evidence being the most named barriers. Interestingly, the skill levels and use of EBP did not appear to be affected by a large number of the respondents not recognising its full definition. Instead, the low use of EBP might be related to the scoring system not fully capturing the integration into everyday clinical practice.

## Data Availability

The datasets and analyses generated in the context of the current study are available from the corresponding author upon reasonable request.

## Appendix

See Table 10.

**Table 10:** International comparison sub-scores [9, 32, 33]

Country (used EBASE for chiropractic)	U.S	Canada	Sweden	Switzerland
Part A (attitude) Median (Mean)	32 (31)	33 (32)	32 (30)	31 (31)
Part B (skill) Median (Mean)	44 (44)	43 (43)	45 (36)	40 (40)
Part D (use) Median (Mean)	8 (10)	8 (6.5)	8 (6)	6 (7)

### Survey

Please note that the survey is not reported in its complete form but only the parts that were analysed and discussed in this work.

#### Part A

On a scale ranging from “strongly disagree” to “strongly agree”, **how would you rate your opinion** on the following statements? (please select the best answer for each category).

#### Part B

On a scale from 1 to 5, with 1 being poor and 5 being advanced, **how would you rate your skills** in the following areas? (please select one per skill area).

#### Part C

Please indicate in **what setting you have received the most in-depth training** in the following areas (please select the best answer for each category). If you select “other”, please write down your highest level of training/education in the space provided.

#### Part D

Please indicate how often you have performed the following activities **over the last month** (please select the best answer for each category).

#### Part E

On a scale ranging from "not a barrier" to major barrier", to what extent do the following factors **prevent you from** participating in evidence-based practice?

#### Part F

On a scale ranging from "not useful" to "very useful", to what extent would the following **strategies** assist you in participating in evidence-based practice?

#### Part G

(please select the best answer to the following questions)

#### Role and Identity

(please select the best answer to the following questions)
